# Gas vesicle-expressing human pluripotent stem cells enable multimodal ultrasound and optical coherence tomographic imaging

**DOI:** 10.1186/s12896-026-01161-x

**Published:** 2026-04-30

**Authors:** John Kim, Alessandro R. Howells, Sumin Park, Xueding Wang, Chengzhi Shi, Xiaojun Lance Lian

**Affiliations:** 1 Department of Mechanical Engineering, University of Michigan, Ann Arbor, MI 48109 USA; 2 Department of Biomedical Engineering, Pennsylvania State University, University Park, PA 16802 USA; 3 Department of Biology, Pennsylvania State University, University Park, PA 16802 USA; 4 The Huck Institutes of the Life Sciences, Pennsylvania State University, University Park, PA 16802 USA; 5 Department of Biomedical Engineering, University of Michigan, Ann Arbor, MI 48109 USA

**Keywords:** Gas vesicles, Human pluripotent stem cells, Ultrasound imaging, Optical coherence tomographic imaging

## Abstract

Genetically encoded imaging reporters are critical tools for tracking cell fate and function in regenerative medicine. Gas vesicles (GVs), air-filled protein nanostructures derived from bacteria, offer unique advantages for noninvasive imaging due to their acoustic and optical properties. In this study, we engineered human pluripotent stem cells (hPSCs) to express GVs using a doxycycline (Dox)-inducible system. Stable GV expression was achieved by TALEN-mediated knock-in of the GvpNtoV cassette at the GAPDH locus together with PiggyBac GvpA integration driven by transposase, followed by antibiotic selection to isolate correctly modified clones. Upon Dox treatment, GVs formed intracellularly and enabled enhanced contrast in both ultrasound and optical coherence tomography (OCT) imaging. Dynamic ultrasound imaging revealed pressure-dependent GV buckling and harmonic signal generation, while OCT imaging confirmed high sensitivity and depth-resolved detection in both in vitro and ex vivo retinal models. Our work establishes a multimodal GV-based reporter platform compatible with human stem cells and clinically relevant imaging modalities. This approach offers a powerful and versatile tool for noninvasively visualizing and tracking therapeutic cells in real time, advancing the development and monitoring of cell-based therapies.

**Supplementary Information:**

The online version contains supplementary material available at 10.1186/s12896-026-01161-x.

## Introduction

Human pluripotent stem cells (hPSCs) [1–3] hold transformative potential for regenerative medicine due to their capacity for unlimited self-renewal and their ability to differentiate into any somatic cell type [4–10]. These characteristics make hPSCs an ideal source for developing cell-based therapies to repair or replace damaged tissues. However, the clinical translation of hPSC-based therapies is significantly limited by the lack of effective tools for noninvasive, real-time monitoring of transplanted cells in vivo. Conventional approaches such as immunohistochemistry and histological analysis provide high-resolution insights into cell fate but are inherently endpoint methods that require tissue collection, limiting their utility for long-term studies.

Current in vivo imaging strategies often rely on optical techniques such as fluorescence [11] or bioluminescence imaging [12]. While these methods are useful for visualizing reporter-labeled cells in small animal models, they suffer from limited tissue penetration and resolution at depth. Consequently, their effectiveness diminishes in larger organisms or clinical settings. To overcome these limitations, ultrasound imaging has emerged as an attractive alternative due to its deep tissue penetration, high spatial and temporal resolution, and established clinical accessibility [13].

In parallel with ultrasound, optical coherence tomography (OCT) has emerged as a powerful, label-free imaging modality capable of providing high-resolution, cross-sectional images of biological tissues at micrometer-scale resolution without ionizing radiation. Originally developed for ophthalmological imaging, OCT has been increasingly adopted in developmental biology to visualize organoid morphogenesis, embryonic tissue formation, and stem cell-derived structures in three dimensions in real time [14, 15]. Despite these strengths, a key limitation of OCT is its reliance on endogenous optical scattering contrast, which often fails to provide sufficient differentiation between cell populations of interest and the surrounding tissue. Genetically encoded contrast agents that modulate optical scattering, such as GVs, offer a promising strategy to address this limitation. Integrating OCT alongside ultrasound, therefore, provides complementary validation. Ultrasound offers deep tissue penetration imaging capability, while OCT delivers superior spatial resolution imaging. This framework not only strengthens the validation of GV expression and functionality, but also positions GVs as versatile reporters across both acoustic and optical imaging platforms.

Various contrast agents have been explored to enhance cell tracking using different imaging modalities [16–19]. Although exogenous microbubble or nanobubble contrast agents have been employed to track cells using ultrasound, they do require external delivery and are transient in nature, thus limiting their long-term reliability [20, 21]. Similarly, nanoparticles have shown promise for cell tracking applications. However, they encounter major challenges in clinical translation due to concerns regarding biocompatibility, clearance pathways, and potential long-term toxicity [22–24].

A breakthrough in this field was the discovery of genetically encodable ultrasound contrast agents. Unlike exogenous contrast agents that require repeated administration or nanoparticles with uncertain clinical feasibilities, genetically encodable reporters offer several distinct advantages. These reporters enable propagation to daughter cells with each cell division, allowing for long-term monitoring without signal dilution [25]. Furthermore, their genetic nature ensures that only viable cells produce the contrast signal, providing functional information beyond localization [26].

These genetically encodable contrast agents come in the form of gas vesicles (GVs) [27]. GVs are air-filled protein nanostructures naturally expressed by certain aquatic bacteria and archaea. It has been shown that GVs enhance ultrasound signals due to their unique acoustic properties [28]. Composed of gas vesicle proteins (Gvp), these nanostructures self-assemble to form intracellular gas-filled compartments that scatter sound waves. Beginning with natural GV-producing organisms, researchers demonstrated that these structures could function as acoustic reporters [28]. Subsequent advances allowed GV expression in *E. coli* [29] and, more recently, within mammalian cells, giving rise to the concept of mammalian acoustic reporter genes (mARGs) [30]. However, until now, mARG expression has been limited to immortalized or cancer-derived cell lines, and their application to primary or therapeutic stem cells has not been demonstrated.

Here, we present the first successful expression of GVs in hPSCs using second-generation mARGs [31], establishing a powerful genetic reporting platform for the most critical cell source in regenerative medicine. The Gvp genes from *Anabaena flos-aquae* can produce 38-fold stronger non-linear acoustic contrast than previously tested clusters when expressed in mammalian cells [31]. We employed a modular gene delivery system combining doxycycline-inducible GvpA expression with constitutive expression of seven accessory Gvp genes (GvpN, GvpJ, GvpK, GvpF, GvpG, GvpW, GvpV, “GvpNtoV”), enabling robust and tunable GV production in hPSCs. Microscopy confirmed intracellular GV formation, and we conducted a series of multimodal imaging validations to demonstrate the functional versatility of this engineered cell line. Specifically, we proved that GV-expressing hPSCs enhance contrast in both ultrasound imaging and optical coherence tomography (OCT) *across both in vitro and ex vivo environments, demonstrating reliable performance even in deep-tissue settings*

By integrating synthetic biology with noninvasive imaging technologies, our work establishes a universal platform for the real-time visualization and longitudinal tracking of therapeutic stem cells, extending beyond the simple provision of a contrast agent … This capability has broad implications for regenerative medicine, enabling dynamic tracking of cell fate, biodistribution, and therapeutic efficacy. Our findings position mARGs as a powerful tool for advancing both basic stem cell research and the development of cell-based therapies.

## Results

### Development and microscopy of mARG hPSCs

**Fig. 1:**
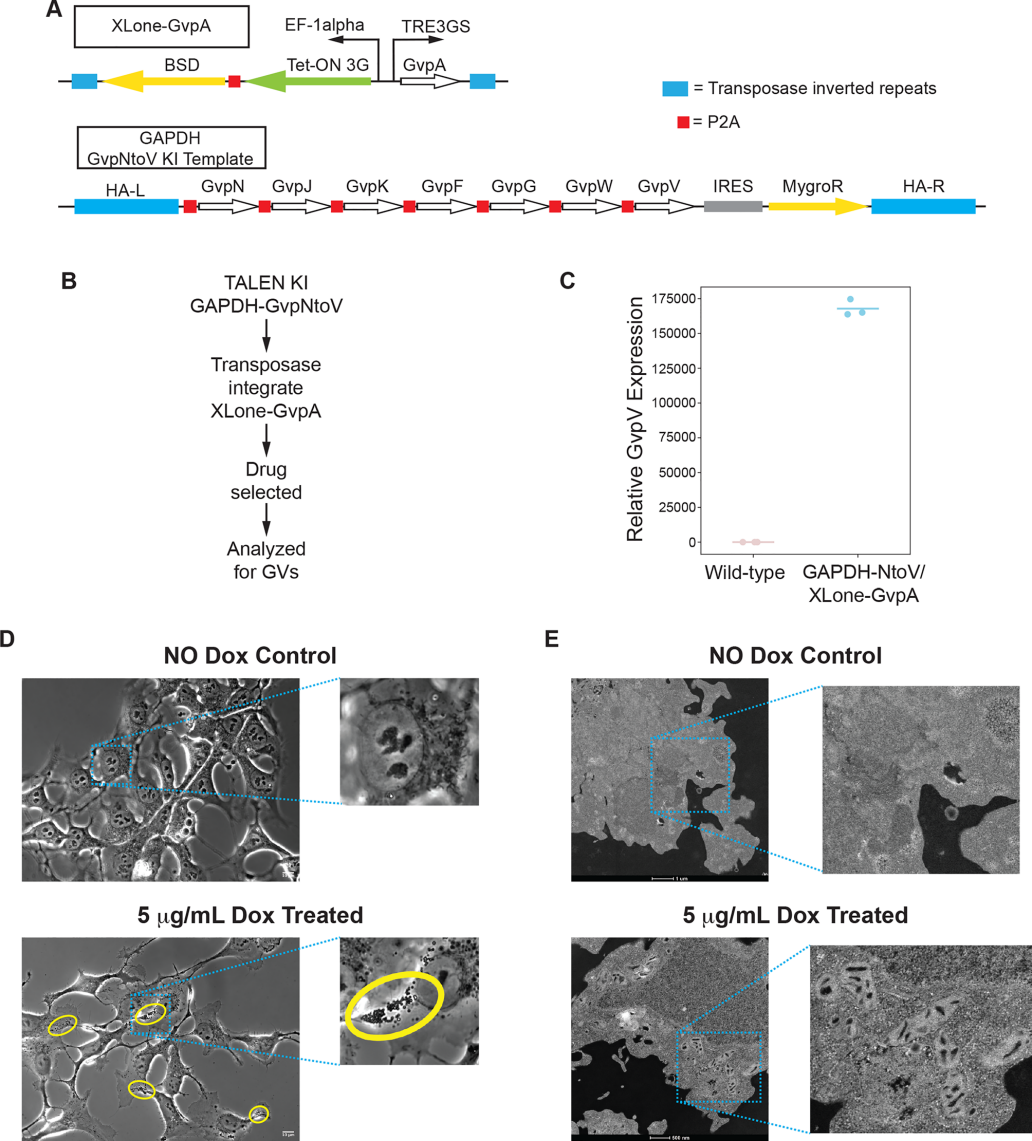
Visualization of GVs within mARG hPscs. **A**) Schematic of our two mARG plasmid designs. Top construct is the Dox inducible, transposable pXlonev0-GvpA cassette, and bottom is the GAPDH-GvpNtoV knock in template. BSD, blasticidin S resistant gene; MygroR, hygromycin B resistant gene; HA-L and HA-R, homology arm left and homology arm right. **B**) Schematic of workflow to generate our drug selected polyclonal mARG hpscs. **C**) qPCR results for relative GvpV expression within wild-type hPscs and our mARG hpsc line. **D**) Representative 60x phase contrast images of cells not treated with Dox (top, No Dox control) and of cells treated with 5 μg/mL dox (bottom). Yellow ovals indicate where GV aggregates are observed. Scale bars = 10 μm. **E**) Representative electron microscope images of cells not treated with Dox (top, No Dox control) and of cells treated with 5 μg/mL dox (bottom). Scale bars = 1 mm and 500 nm

To engineer hPSCs for GV expression, we utilized the GV gene cluster from *A. flos-aquae*. Specifically, the primary structural protein gene GvpA was cloned into our Dox-inducible XLone vector (Fig. 1A), while the remaining seven accessory genes (GvpNtoV) were inserted into a glyceraldehyde-3-phosphate dehydrogenase (GAPDH) locus via transcription activator-like effector nuclease (TALEN)-mediated knock-in (Fig. 1A). This design ensures robust constitutive expression of the accessory genes via a knock-in downstream of GAPDH gene, minimizing the likelihood of silencing, and permits precise Dox-dependent induction of GvpA expression through the integrated Tet-On 3 G system present in the XLone vector. Moreover, the incorporation of distinct drug resistance markers, blasticidin resistance in the XLone construct and hygromycin resistance in the GAPDH donor vector, facilitates effective selection of the engineered cells.

Following TALEN mediated knock-in of GvpNtoV and the transposase-mediated integration of XLone-GvpA into H1 hPSCs, we selected a polyclonal population using the corresponding drugs (blasticidin and hygromycin) (Fig. 1B). Validation by quantitative PCR (qPCR) revealed that relative GvpV RNA levels in the engineered cells (mARG hPSCs) were approximately 170,000-fold higher than those in wild-type H1 cells, confirming successful integration and selection (Fig. 1C).

We further demonstrated GV formation using microscopy. Phase contrast imaging at 60x magnification of mARG hPSCs treated with 5 mg/mL Dox for 72 hours revealed cytoplasmic structures indicative of GV aggregates, in contrast to the clear cytoplasm observed in untreated mARG hPSCs (Fig. 1D). Additionally, electron microscopy demonstrated that while untreated control cells maintained normal cytoplasmic appearance, Dox-treated mARG hPSCs exhibited distinct, gas-filled vesicular structures, likely representing cross sections of the GVs (Fig. 1E).

### Gas vesicles as a potential contrast agent for static ultrasound imaging

**Fig. 2:**
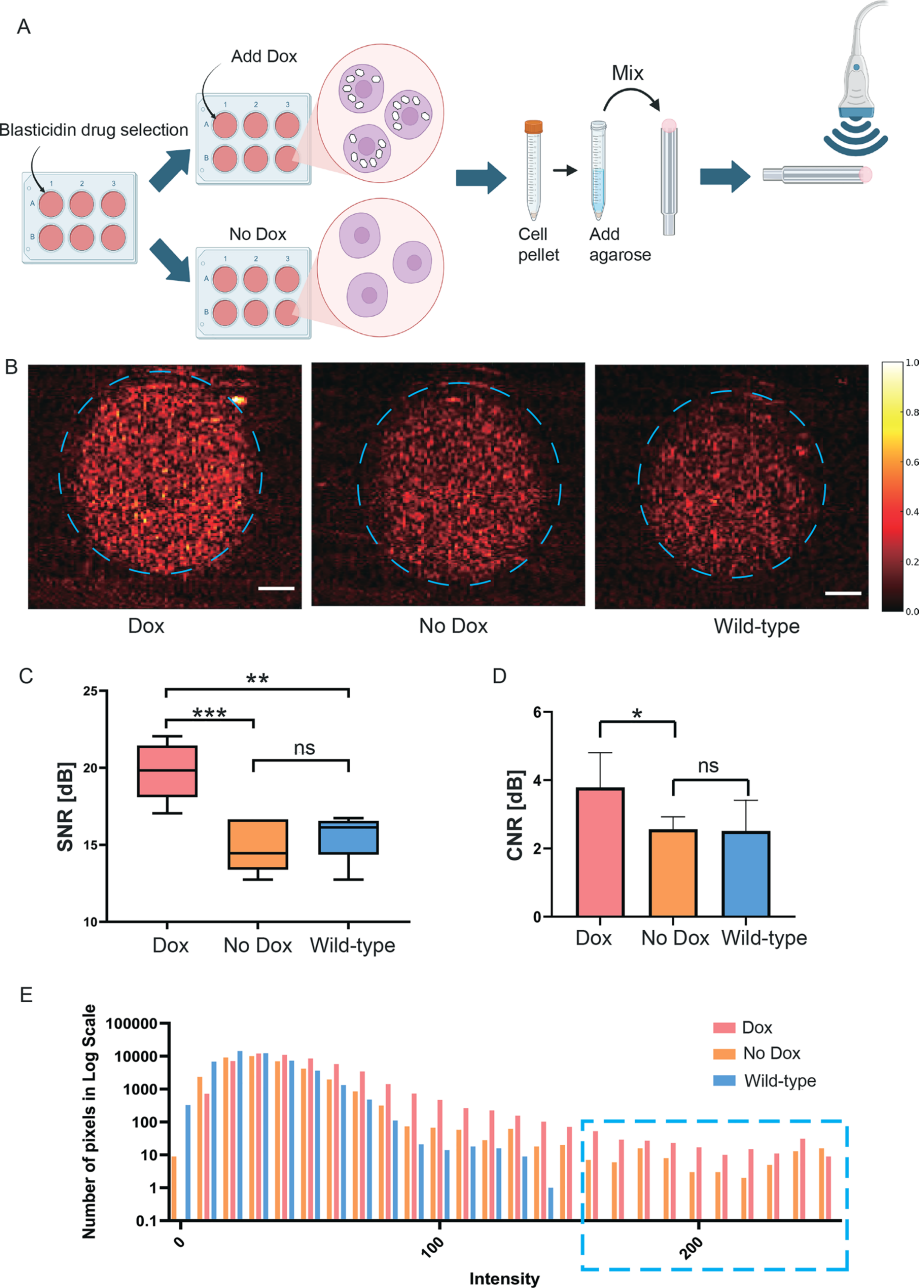
Static ultrasound images of GV-expressing hPscs. **A**) Schematic drawing of static imaging set up that shows from drug selectable GV expressions on cells to imaging. **B**) Ultrasound cross section images of cell samples mixed with 1% agarose gel. The hot-scale B-mode images present the Dox treated mARG hPscs (left), the non-doxycycline treated mARG hpscs (middle), and wild-type stem cells (right). Color scale: 1 × 10 ^3^–1 × 10 ^5^. **C**) SNR values for different conditions in decibels (dB). The Dox-treated samples exhibit significantly higher SNR compared to No Dox and wild-type groups (**p < 0.01, ***p < 0.001, ns: not significant). **D**) CNR values in dB for each condition. The Dox-treated samples demonstrate the highest CNR, indicating enhanced contrast between the GV expressing cells and the background. (*p < 0.05) **E**) Histogram (log scale) represents the distribution of pixel intensities. Dox-treated samples exhibit a higher number of high-intensity pixels compared to No Dox and wild-type samples, as highlighted in the dashed blue box. Scale bars = 1 mm. Color scale represents IQ data acquired from Verasonics

To demonstrate the feasibility of using GVs as contrast agents in ultrasound imaging, we used our established mARG hPSCs prepared with or without Dox induction, alongside wild-type H1 hPSCs to serve as a control. We first performed simplified static imaging, without using high intensity ultrasound to induce GV buckling [30, 32] (Fig. 2A). We hypothesized that a concentration of GV-containing hPSCs would scatter more ultrasound signal as a cluster due to the acoustic impedance mismatch between the gas content in GVs and the fluids in the cytoplasm, even when the vesicles are sub-wavelength in size.

Ultrasound imaging of Dox-treated hPSC clusters revealed pronounced contrast in B-mode imaging, observable even prior to background subtraction (Fig. 2B). Quantitative analysis of the signal-to-noise ratio (SNR) indicated a marked difference between Dox-treated cells and untreated or wild-type cells. The SNR, quantified for each condition, demonstrated that Dox-treated mARG hPSCs exhibited a significantly higher SNR (19.7 ± 1.9 dB) compared to the untreated (14.9 ± 1.7 dB) and wild-type (15.6 ± 1.6 dB) cells, thereby confirming that GV expression contributes to enhanced ultrasound signal (Fig. 2C). The minimal difference between untreated and wild-type cells further substantiates that in the absence of Dox, GV expression does not occur, demonstrating the extremely low background expression of XLone without Dox [33]. The faint baseline echogenicity in wild-type and no Dox controls originates from intrinsic acoustic scattering by dense cellular structures, which create a slight acoustic impedance mismatch with the surrounding agarose matrix [13]. However, the expression of gas-filled GVs in Dox-treated hPSCs substantially amplifies this mismatch, yielding a hyper-echogenic response that clearly overshadows the background cellular scattering [28, 30, 32].

Additionally, contrast-to-noise ratio (CNR) values were calculated to assess the delineation of the GV-expressing region from the surrounding background. As expected, the Dox-treated mARG hPSCs exhibited the highest CNR (3.8 ± 1.0 dB). However, the differences among the groups (Dox, No Dox, Wild-type) were less pronounced compared to those observed in the SNR analysis, suggesting that while GV expression improves ultrasound contrast, the absolute signal strength (as evaluated by SNR) serves as a more robust indicator of GV presence (Fig. 2D). A histogram on a logarithmic scale of pixel intensity distribution across the different treatment conditions further highlighted the presence of high-intensity pixels in the Dox-treated mARG hPSCs (Fig. 2E). Specifically, the highlighted blue box in the histogram indicates a greater number of high-intensity pixels in the Dox-treated group, consistent with enhanced signal output from GVs, while untreated and wild-type samples demonstrated a lower and more uniform intensity distribution (Fig. 2E).

While these in vitro results confirmed the basic acoustic functionality of GVs in hPSCs, we further evaluated whether this contrast enhancement is maintained in deep tissue environments where acoustic attenuation is significant. By performing ex vivo ultrasound imaging using a chicken breast tissue phantom, we confirmed that GV expressing hPSCs (Dox-treated) provide robust contrast enhancement at depths of approximately 16 mm. The Dox group maintained a higher average SNR (14.5 dB) compared to the wild-type (10.6 dB) and No Dox (10.2 dB) groups, demonstrating that the acoustic backscattering of GVs has meaningful signal specificity for organ monitoring (Supplementary Fig. S1).

### Dynamic imaging of GVs in mARG hPSCs

We previously demonstrated that a drug selection strategy can effectively isolate a mixed population of GV-expressing HEK293 cells [32]. In that study, we applied focused ultrasound (FUS) at a mechanical index below the FDA limit of 1.9 [34]. Mechanical index is a parameter that reflects the potential for acoustic cavitation and the corresponding bioeffects. At this low level, we successfully induced GV buckling without causing any acoustic harm to the cells. Moreover, by synchronizing the FUS-induced GV buckling with a B-mode imaging system, we were able to capture dynamic ultrasound contrast seamlessly.

**Fig. 3:**
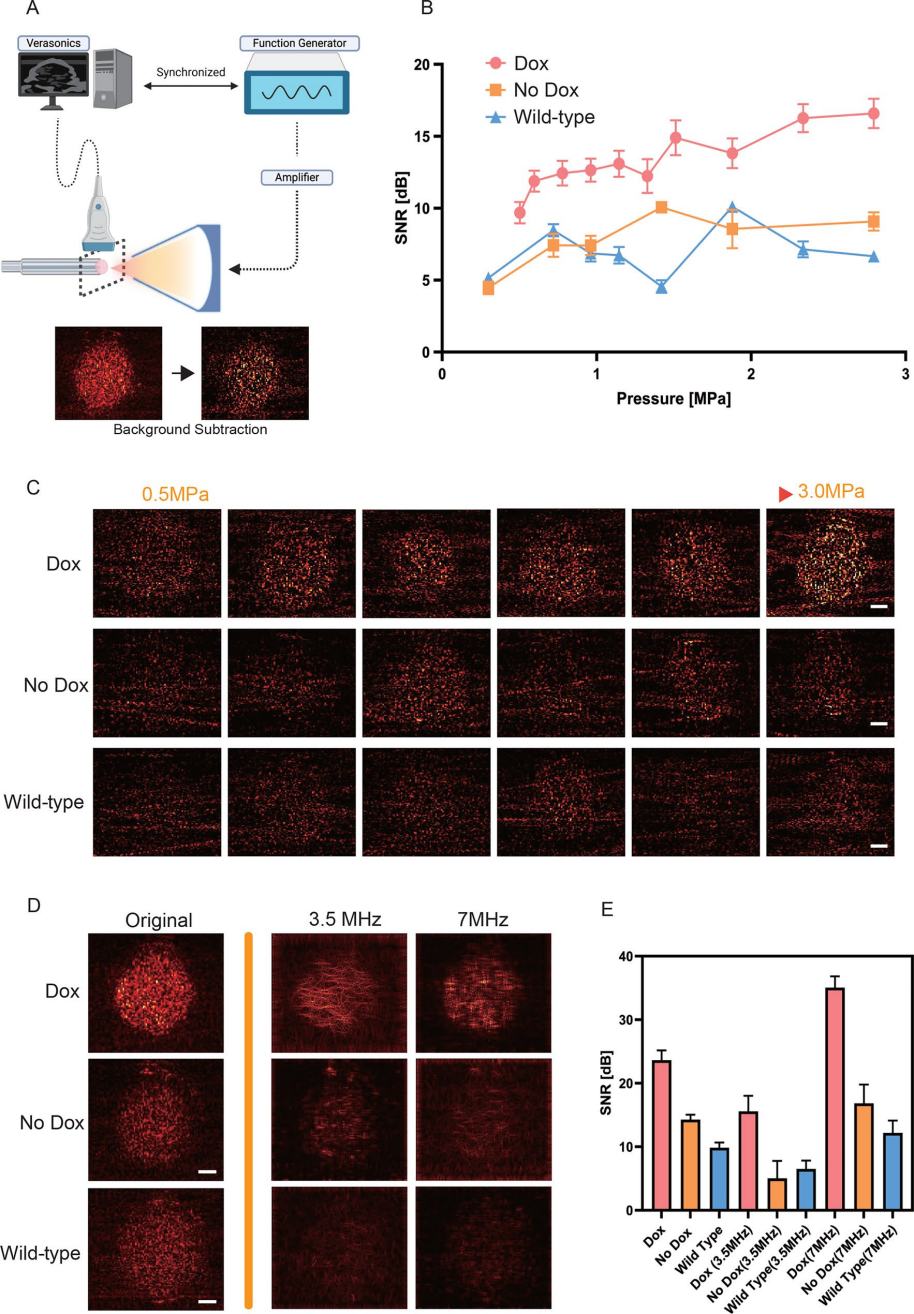
Dynamic ultrasound images of GV-expressing hPscs. **A**) the schematic illustrates the experimental setup for FUS stimulation and subsequent imaging that are synchronized. Ultrasound response was analyzed after background subtraction to enhance signal detection. **B**) the SNR values of the region of interest (ROI) are plotted against increasing ultrasound pressure (0–3 MPa) for three sample conditions (Dox, No Dox, wild-type). Dox-treated samples exhibit a continuous increase in SNR with increasing pressure, whereas No Dox and wild-type samples show significantly lower and more variable SNR responses. **C**) a series of ultrasound images demonstrates the effect of increasing FUS pressure (0.5 MPa to 3.0 MPa) on different sample conditions. Dox-treated samples show a progressive enhancement in brightness and signal intensity, particularly at higher pressures, suggesting GV buckling. Color scale: 1e ^3^–1e ^5^. **D**) ultrasound images were processed with frequency-based filtering (3.5 MHz and 7 MHz) to enhance harmonic signal detection. Dox-treated samples show distinct harmonic components at 3.5 MHz and 7 MHz, while No Dox and wild-type samples display weak or no discernible harmonic signals. Color scale: (original) 1e ^3^–1e ^5^ /(3.5 and 7 MHz): 1e ^3^–3e ^4^. **E**) the bar graph quantifies SNR values for the original and frequency-filtered images. Dox-treated samples exhibit significantly higher SNR across all conditions, particularly in the 3.5 MHz and 7 MHz filtered images, reinforcing the role of GVs in enhancing ultrasound contrast. No Dox and wild-type cells maintain consistently lower SNR values, indicating the absence of detectable GV activity. Scale bars = 1 mm. Color scale represents IQ data acquired from Verasonics

Building on our previous static ultrasound imaging setup, we oriented the FUS vertically to enable simultaneous GV buckling and real-time imaging, thereby optimizing detection, sensitivity, and spatial resolution (Fig. 3A). Unlike in static imaging, we applied post-processing to the captured frames to delineate changes following FUS exposure (Fig. S1A). Specifically, we subtracted post-FUS frames from pre-FUS frames to visualize GV-driven dynamic contrast. The input peak negative pressures ranged from 0.5 to 3 MPa and corresponded to a mechanical index below 1.2 (Fig. S1B).

Under these conditions, the SNR of Dox-treated mARG hPSCs steadily increased with increasing pressure, ultimately plateauing at higher pressures (Fig. 3B). This result indicates that GV buckling enhances the acoustic response, although it is constrained by the concentration of GVs. By contrast, both the No Dox and wild-type cells maintained significantly lower SNR values across all pressures tested. Notably, the disparity between the Dox-treated and control groups became more pronounced at pressures above 1.5 MPa, suggesting that GV-containing cells are more susceptible to acoustic cavitation and potential cellular damage [35, 36].

Overall, these findings confirm that GV expression markedly enhances ultrasound contrast in a pressure-dependent manner, while cells without GV expression are largely unaffected (Fig. 3B, C). To further boost harmonic signal detection, ultrasound images were processed using frequency-based filtering at 3.5 MHz and 7 MHz. Dox-treated mARG hPSCs exhibited strong harmonic components at both frequencies, whereas the No Dox and wild-type samples showed weak or undetectable harmonic signals (Fig. 3D, E). The bar graph quantifies SNR values for both original and filtered images, revealing that Dox-treated samples consistently achieved significantly higher SNRs (23.6 ± 1.5 dB), especially in the frequency-filtered images (3.5 MHz: 15.6 ± 2.4 dB; 7 MHz: 35.1 ± 1.8 dB). By contrast, the No Dox and wild-type cells retained lower SNR values, underscoring the absence of detectable GV activity.

### In vitro and ex vivo OCT imaging of GVs in mARG hPSCs

**Fig. 4:**
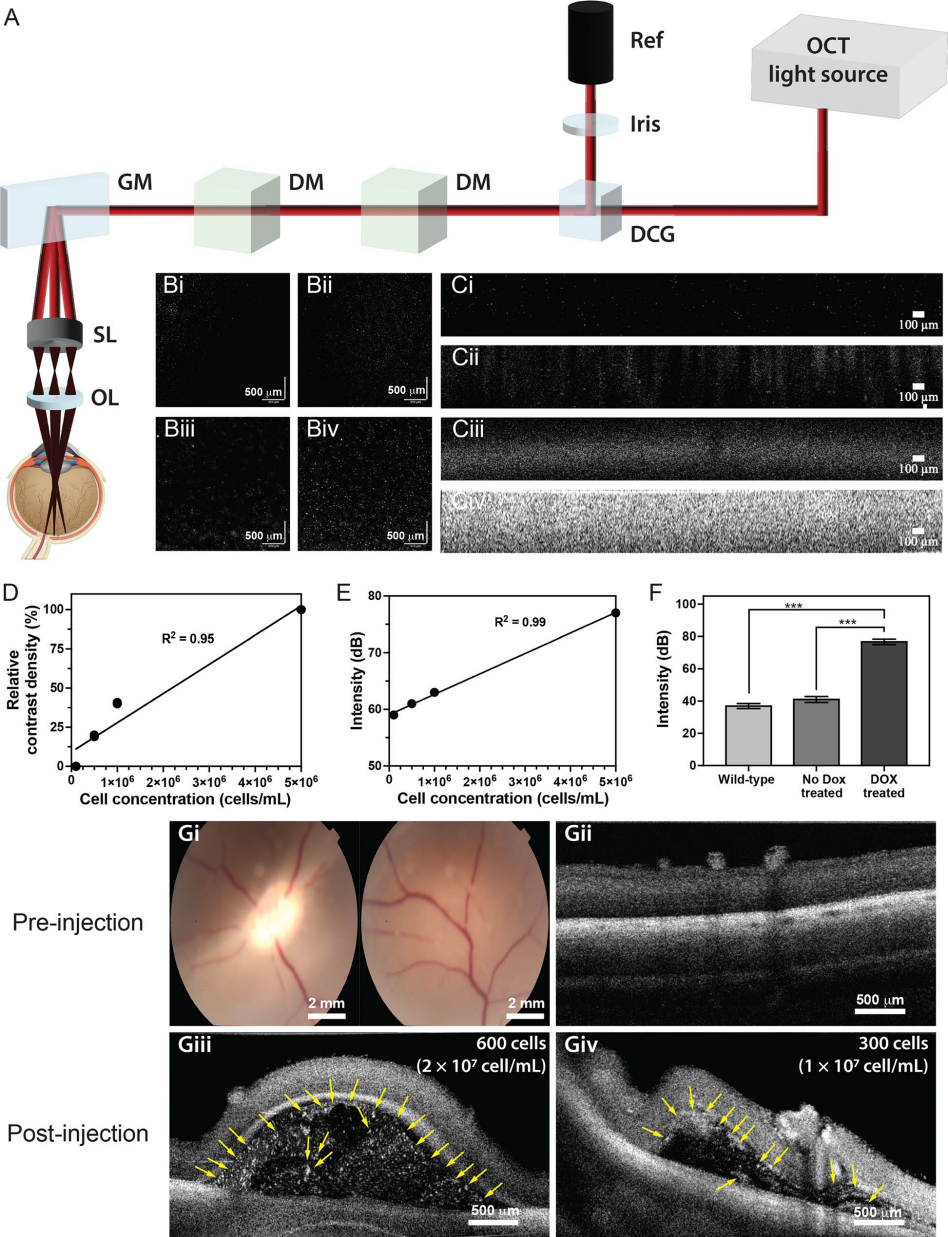
OCT of GV-expressing stem cells. **A**) Schematic diagram of the OCT system used for imaging GV-expressing hPscs. (ref reference arm; DCG dispersion compensation glass; DM dichroic mirror; GM galvanometer; SL scan lens; OL ophthalmic lens). Representative OCT images of agarose phantoms with GV-expressing hPscs: Bi – biv) en-face images (scale bar = 500 mm) and ci – Civ) B-scan images at concentrations (scale bar = 100 mm) of (**i**) 1 × 10 ^5^ cells/mL, (ii) 5 × 10 ^5^ cells/mL, (iii) 1 × 10 ^6^ cells/mL, and (iv) 5 × 10 ^6^ cells/mL, respectively. Quantitative analysis: D) relative signal density versus cell concentration (R^2^ = 0.95), and **E**) Signal intensity versus cell concentration (R^2^ = 0.99). **F**) Intensity comparison among wild-type, no-Dox-treated, and Dox-treated in vitro samples. **G**) ex vivo imaging of porcine eye: gi) fundus photograph showing a healthy optic disc and retinal vasculature before injection. Scale bar = 2 mm. Gii) B-scan OCT image of the porcine retina before injection. Scale bar = 500 mm. OCT image post-injection of giii) 600 cells at a concentration of 2 × 10 ^7^ cells/mL, and giv) 300 cells at a concentration of 1 × 10 ^7^ cells/mL. The yellow arrows indicate GV-expressed individual stem cells. Scale bar = 500 mm

GVs can serve as effective OCT contrast agents [37]. To assess their detectability when expressed in hPSCs, we developed a custom-modified SD-OCT system for both in vitro phantom and ex vivo ocular imaging (Fig. 4A). This system offers high sensitivity alongside depth-resolved imaging capabilities.

For in vitro validation, we prepared four sets of agarose phantoms containing Dox-treated mARG hPSCs across a range of cell concentrations. OCT images of these phantoms revealed a uniform cell distribution within the agarose matrix. En-face images displayed progressively higher signal densities with increasing cell concentrations (Fig. 4Bi-Biv), while B-scan images provided detailed depth-resolved information (Fig. 4Ci-Civ, Supplementary video 1–4). These observations confirm the system’s sensitivity, highlighting the enhanced contrast generated by GV-expressing mARG hPSCs. Quantitative analyses (Fig. 4D–E) further revealed a strong linear correlation between cell concentration and both signal density (R^2^ = 0.95) and peak intensity (R^2^ = 0.99), with significantly increased values at higher cell concentrations.

To confirm specificity, we compared phantoms embedded with wild-type hPSCs and untreated mARG hPSCs exhibited substantially lower signal intensities (wild-type: 36.9 ± 1.5 dB, untreated: 41.0 ± 1.8 dB) compared to the Dox-treated cells (76.8 ± 1.7 dB), confirming that the enhanced signals are specifically attributable to GVs (Fig. 4F). The DOX-treated cells indicated around 1.8 - 2 times higher signal intensities than wild-type and untreated cells.

Building on the robust in vitro imaging results, we conducted ex vivo feasibility of monitoring mARG hPSCs within the porcine eye model. We prepared fundus photographs and OCT images of an ex vivo porcine eyeball (Fig. 4G). The pre-injection fundus photograph clearly depicts the optic disc and retinal vasculature, establishing a healthy baseline (Fig. 4Gi). A baseline B-scan OCT image of the retina further establishes a reference for subsequent imaging comparisons (Fig. 4Gii). Following subretinal injection of GV-expressing mARG hPSCs at concentrations of 2 × 10^7^ cells/mL and 1 × 10^7^ cells/mL, OCT images revealed distinct hyper-reflective signals localized in the subretinal space (Fig. 4Giii-Giv). Quantitative analysis indicated that while the retinal layer exhibited a mean intensity of 46.6 ± 2.2 dB, the one injected GV-expressing mARG hPSCs generated a significantly higher intensity of 59.8 ± 2.3 dB, which corresponding to a 128% enhancement in signal contrast relative to the surrounding tissue.

Collectively, these findings demonstrate the feasibility of imaging and tracking GV-expressing stem cells using OCT, highlighting their promising applications in tissue engineering, regenerative medicine, and ophthalmology.

## Discussion

In this study, we demonstrate the successful engineering of hPSCs to express GVs for multimodal imaging applications. By leveraging a Dox-inducible system for GvpA expression and constitutive expression of the remaining GV structural genes, we achieved robust and controllable GV production in hPSCs. Microscopy data confirmed the presence of intracellular gas-filled vesicles upon induction, validating our genetic design. Importantly, the use of dual antibiotic selection helped ensure integration fidelity and enabled efficient enrichment of GV-positive hPSCs, offering a streamlined workflow for producing reporter stem cell lines.

The ability of GV-expressing hPSCs to enhance contrast in static and dynamic ultrasound imaging was clearly demonstrated. Cells with Dox treatment produced significantly higher SNR and CNR ratios compared to controls, indicating successful acoustic modulation by intracellular GVs. Under FUS stimulation, we observed pressure-dependent buckling behavior, a hallmark of functional GV activity, as well as harmonic signal generation. Importantly, the observed pressure-dependent phase transitions in GVs enabled both enhanced imaging contrast and the potential for cavitation-mediated modulation, expanding their functional utility beyond passive contrast enhancement. Notably, these dynamic responses were absent in wild-type and cells without Dox treatment, reinforcing the specificity and reliability of GV expression as an imaging marker.

We further extended our findings by applying OCT, where GV-expressing stem cells showed strong signal intensities both in vitro and in ex vivo porcine retina models. Quantitative analyses demonstrated a clear correlation between cell concentration and OCT signal strength, establishing the sensitivity of this imaging platform. In the ex vivo retinal setting, injected cells remained detectable with enhanced contrast relative to the surrounding tissue, albeit at slightly reduced levels compared to in vitro results. These data suggest that while GVs are effective optical scatterers, physiological factors in tissue may attenuate signal propagation and highlight the need for further optimization under in vivo conditions. The OCT system employed in this study achieves an axial resolution of approximately 5.5 µm and lateral resolution of approximately 13.0 µm, which is sufficient to resolve individual hPSCs given their typical diameter of 10.0 to 18.0 µm [38]. However, individual GV nanostructures, which are nanometer in diameter, fall far below the diffraction limit of OCT and cannot be directly visualized as discrete objects. The OCT contrast enhancement observed in GV-expressing hPSCs therefore reflects a population-level increase in optical backscattering, arising from the refractive index mismatch between the gas-filled protein shells of GVs and the surrounding tissue, rather than single-vesicle resolution. It should be noted that in this study, OCT and ultrasound imaging were employed as independent feasibility assessments. Each modality was used separately to evaluate whether GV-expressing hPSCs generate detectable contrast under that specific imaging platform. Several limitations of OCT-based GV detection remain to be addressed. The specificity of OCT contrast may be constrained by the difficulty of distinguishing GV-derived backscattering from other highly scattering intracellular components such as lipid or organelle-dense regions. In addition, in densely packed three-dimensional cell constructs, multiple scattering effects may attenuate OCT signal with depth, limiting detection sensitivity in deeper regions. Further studies employing advanced OCT approaches may improve detection specificity by exploiting the unique optical scattering characteristics of gas-filled nanostructures. Building on these findings, to further enhance the sensitivity of OCT in imaging the GV-expressing hPSCs in ophthalmic tissues, increase the optical scattering contrast generated by the GVs in these cells will be a key focus of our future research. Such advancements would establish GV-expressing hPSCs as a platform tool for understanding cell-based therapies in preclinical and clinical settings in ophthalmology.

Collectively, our work establishes GVs as genetically encoded, multimodal contrast agents compatible with ultrasound and OCT imaging in stem cells. This platform holds significant promise for noninvasive tracking of therapeutic cells in regenerative medicine and real-time monitoring of cell-based therapies. Future work will focus on optimizing GV expression to enhance signal robustness in vivo, evaluating long-term stability of the reporter system, and extending this strategy to additional cell types and disease models. Integration of GV-based imaging into clinical workflows could transform our ability to monitor cellular dynamics with high spatial and temporal resolution.

## Materials and methods

### Plasmid cloning

All plasmids were cloned using the Takara In-Fusion cloning kit. Primers were designed by the Takara In-Fusion online portal. For XLone-GvpA, XLone-GFP (Addgene # 96,930) was restriction enzyme digested with SpeI and KpnI. The GvpA insert was PCR amplified from a plasmid gifted to us from Dr. Mikhail Shapiro. For the GAPDH-GvpNtoV plasmid, pUC-GW-Amp backbone was digested with MluI and AscI. GAPDH homology arms and IRES-MygroR were PCR amplified from pGAPTrap-mCherry-IRESMygro (Addgene # 82,505) and GvpNtoV were PCR amplified from a plasmid gifted to us from Dr. Mikhail Shapiro. After 10 mL In-Fusion reaction, 5 mL were transformed into Stbl3 Chemically Competent *E. coli* following manufacturing protocol. E.coli were plated onto Ampicillin selection plates and cultured overnight. Clones were then picked and cultured for 18 hours in LB broth supplemented with Ampicillin. Finally, plasmids were harvested via Zymopure plasmid miniprep kit and sequenced.

### hPSC maintenance and genetic engineering

hPSCs were cultured on iMatrix-511 coated Falcon well plates, in either essential 8 or mTeSR1 media. Routine passages were performed once confluency reached about 70% by dissociating with accutase and centrifuging for 4 minutes at 200 RCF. Cell pellets were then resuspended in media containing ROCK inhibitor and plated onto freshly coated wells. For TALEN mediated knock-in of GvpNtoV at the GAPDH locus, wild-type H1 hPSCs were pelleted and resuspended in 100 mL PBS buffer containing 10 mg of our donor vector, 5 mg of pGAPTrap-TALEN 1 (Addgene #83368), and 5 mg of pGAPTrap-TALEN 2 (Addgene #83369). Cells were then transferred to a cuvette and nucleofected on program CB150 on a Lonza 4D Nucleofector. Cells were then plated at high density and after expansion, were drug selected to a final concentration of 200 mg/mL hygromycin B. For XLone-GvpA integration, the same nucleofection protocol was followed, however they were nucleofected with 5 mg XLone-GvpA and 5 mg EF1a-hyPBase transposase plasmids, and were drug selected to a final concentration of 20 mg/mL BSD.

### Quantitative polymerase chain reaction (qPCR)

RNA was collected from hPSC samples using the Direct-zol RNA MicroPrep kit (Zymo Research) according to the manufacture’s instructions. cDNA synthesis was then carried out on 500 ng RNA using the ZymoScript RT Premix (Zymo Research). cDNA was then diluted to 2 ng/μL. qPCR reactions were then set up in triplicate with 10 μL Power SYBR Green Master Mix (ThermoFisher), 1 μL cDNA (2 ng/μL stock), 8 μL molecular biology grade water, and 1 μL primer mix (5 μM stock). Forward GvpV primer used was TGTACAAGATGGTCACCGAGAA and reverse GvpV primer was TGTTGCTTTCCACGAAGATGTT. Housekeeping gene used for normalization was GAPDH, (forward: GTGGACCTGACCTGCCGTCT and reverse: GGAGGAGTGGGTGTCGCTGT). qPCR reactions were then run on the CFX Connect Real-Time System (Bio Rad) with an initial 10-minute incubation at 95 °C and 40 cycles of 15 seconds at 95 °C followed by 75 seconds at 60 °C. Relative GvpV expression was then calculated based on Cq values and normalized to GAPDH.

### Phase contrast imaging and electron microscopy

Phase contrast was performed by plating cells sparsely onto glass bottom wells. Cells were then cultured for a total of 72 hours, either with or without 5 mg/mL Dox, and the day prior to imaging, media was changed containing ROCK inhibitor. Phase contrast images were acquired using a custom-built microscope based on an Olympus IX-73 body, equipped with Olympus phase contrast objectives, a Kiralux 12.3 MP monochrome CMOS camera (CS126MU, Thorlabs), a 100W halogen lamp (Olympus), and a SOLA U-nIR light engine (Lumencor).

For electron microscopy, about 1 × 10^6^ cells were fixed with 2.5% glutaraldehyde/4% Paraformaldehyde and post fixed with 1% Osmium Tetroxide in 0.1 M Sodium Cacodylate Buffer. Sample were then stained with uranyl acetate, dehydrated with increasing concentrations of ethanol, and embedded in Epon Epoxy. Grids were then imaged using scanning electron microscopy and transmission electron microscopy (S/TEM Talos F200×2 from Thermo Scientific).

### Sample preparation for ultrasound imaging

Two 6-well plates were prepared for each experiment to acquire samples for ultrasound imaging. Before ultrasound experiment, stem cells were treated with doxycycline for 72 hours to assure abundant time for GVs to fully grow. The cells were dissociated with Accutase, and counted with Cell counting device (Cell Counter 3 FL, ThermoFisher) at a concentration of 2 × 10^7^ cells/ml. The harvested cells were centrifuged and resuspended in 1% agarose gel (ThermoFisher) to create a solidified sample within a plastic holder, ensuring a stationary position in a water tank filled with deionized, degassed water at 37 °C. A volume of 200 mL of the cell-gel mixture was layered on top of the holder, allowing the cells to not only fill the holder but also form an additional layer above it. Such method allowed optimal transducer alignment, minimizing imaging of the outer perimeter of the plastic sample that could otherwise introduce sound artifacts.

*Ex vivo chicken breast tissue phantom preparation*: To evaluate deep tissue contrast enhancement, an ex vivo chicken breast phantom was prepared. A custom sample holder was constructed by replacing two opposite walls of a standard cuvette with an acoustically transparent membrane. To facilitate easy localization during imaging (Supplementary Fig. 1), the sample was cast inside the cuvette as two distinct layers: a blank agarose gel and a cell-agarose mixture at a concentration of 1 × 10^6^ cells/ml. This modified cuvette was then inserted under fresh chicken breast tissue at a depth of approximately 16 mm. Finally, the ultrasound transducer was coupled to the top surface of the tissue using acoustic gel.

*Imaging transducer*: For ultrasound imaging, Vantage NXT(Verasonics) was used to collect data, employing transducer a L22-8 v for in vitro experiments and transducer a L22-14vX for ex vivo studies. The transducer was positioned vertically to capture the cross-sectional area of the cell-agarose phantom. The center frequency was set to 15.6 MHz for in vitro imaging and 18 MHz for ex vivo imaging, with sampling rate at 200% of the Nyquist frequency. For dynamic ultrasound imaging acquisition, three angles with three apertures were utilized to generate each frame, resulting in a total of nine transmit-receive pairs per image. The interval between each event was 200 ms, achieving a frame rate of approximately 800 frames per second

*Focused ultrasound*: A single-element focused ultrasound transducer operating at 3.5 MHz (H-101, SonicConcepts) was fitted with a coupling cone backfilled with degassed, deionized water such that the focus point is at the tip of the coupling cone. The structural input of FUS where it is synchronized to Verasonics, sending out signals and amplified at 3.5 MHz. FUS transducer was connected to both function generator and amplitude so that it sends out 10 cycles of desired input voltage only when triggered by imaging system. The synchronized set up assured the repeatability and consistence of experimental outcome

*Post Processing*: Post-processing was performed to enhance signal detection, quantify ultrasound contrast, and evaluate GV activity under FUS stimulation. Ten pre-collapse frames and forty post-collapse frames were captured per 10-cycle burst sine wave. Ten pre-frames were averaged to be subtracted from the post frames images. Among the subtracted images, SNR and CNR was collected from region of interest (ROI). ROI was defined as same as static image.

$${\rm{CNR }} = {\rm{ }}{{{{\rm{\mu }}_{{\rm{ROI}}}} - {{\rm{\mu }}_{{\rm{Background}}}}} \over {{{\rm{\sigma }}_{{\rm{Background}}}}}}$$

$${{\rm{\mu }}_{{\rm{ROI}}}}{\rm{ }} = {\rm{ }}{1 \over {{\rm{MN}}}}\mathop \sum \limits_{{\rm{i}} = 1}^{\rm{M}} \mathop \sum \limits_{{\rm{j}} = 1}^{\rm{N}} {\rm{Signa}}{{\rm{l}}_{{\rm{ROI}}}}\left( {{\rm{i}},{\rm{ j}}} \right)$$

$${{\rm{\mu }}_{{\rm{Background}}}}{\rm{ }} = {\rm{ }}{1 \over {{\rm{MN}}}}\mathop \sum \limits_{{\rm{i}} = 1}^{\rm{M}} \mathop \sum \limits_{{\rm{j}} = 1}^{\rm{N}} {\rm{Signa}}{{\rm{l}}_{{\rm{Background}}}}\left( {{\rm{i}},{\rm{ j}}} \right)$$

$${\rm{SNR }} = {\rm{ }}10{\rm{log}}\left( {{{{{\rm{I}}_{{\rm{ROI}}}}} \over {{{\rm{I}}_{{\rm{Background}}}}}}} \right)$$

$${{\rm{I}}_{{\rm{ROI}}}}{\rm{ }} = {\rm{ }}{1 \over {{\rm{MN}}}}\mathop \sum \limits_{{\rm{i}} = 1}^{\rm{M}} \mathop \sum \limits_{{\rm{j}} = 1}^{\rm{N}} {\rm{Signal}}_{{\rm{ROI}}}^2\left( {{\rm{i}},{\rm{ j}}} \right)$$

$${{\rm{ I }}_{{\rm{Background}}}}{\rm{ }} = {\rm{ }}{1 \over {{\rm{MN}}}}\mathop \sum \limits_{{\rm{i}} = 1}^{\rm{M}} \mathop \sum \limits_{{\rm{j}} = 1}^{\rm{N}} {\rm{Signa}}{{\rm{l}}^2_{{\rm{Background}}}}\left( {{\rm{i}},{\rm{ j}}} \right)$$

In these equations, M and N are the dimensions of the ROI and background in pixels, *Signal*_ROI_*(i,j)* and *Signal*_Background_*(i,j)* are the signal intensity at pixel*(i,j)* within the ROI and background area, respectively. *I*_ROI_ is the average signal intensity of the ROI, *Signal*_ROI_*(i,j)* is the average background signal intensity.

### Sample preparation for OCT imaging

*Agarose phantom preparation*: To mimic the optical and mechanical properties of biological tissues and ensure reproducible and controlled experimental conditions, we used a 1% w/v agarose gel (Sigma Aldrich, St. Louis, MO, USA) in this study [37, 39]. Agarose powder was dissolved in deionized water by heating to 90 °C under constant stirring until fully homogenized. The solution was degassed and cooled to 37 °C to ensure uniformity and minimize bubble formation. GV-expressing cells were centrifuged, resuspended in the agarose solution at a predefined concentration, and cast into a customized mold. The gel solidified within minutes. The optical transparency and acoustic impedance of the agarose phantom closely resembled those of biological soft tissues, making it suitable for calibration and validation of imaging systems.

*Porcine eyeball preparation*: For ex vivo validation of OCT contrast enhancement, freshly excised porcine eyeballs were used as the tissue model. The porcine eye was selected for several reasons. Anatomically and optically, the porcine eye closely resembles the human eye in terms of dimensions, tissue layer organization, and refractive properties, making it a well-established and translationally relevant model for OCT imaging studies [40–42]. The vitreous humor provides a structurally homogeneous, low-scattering background medium that minimizes intrinsic OCT signal, thereby enabling sensitive detection of contrast contributions arising specifically from GV-expressing cells introduced into the tissue. In addition, porcine eyes are obtainable as byproducts of food processing, and readily available, offering a practical and ethically favorable tissue source that does not require dedicated animal experiments. Therefore, these properties make the porcine eyeball an appropriate model for benchmarking the OCT contrast performance of GV-expressing hPSCs in a tissue-mimicking environment. Fresh porcine eyeballs were obtained from a local slaughterhouse, where all animals were euthanized on the same day as the study to preserve tissue hydration and structural integrity. The eyeballs were transported at 4 °C in Dulbecco’s Modified Eagle’s Medium (DMEM; Gibco, Waltham, MA, USA) supplemented with 1% penicillin and streptomycin (ThermoFisher, Waltham, MA, USA), 1% L-glutamine (Gibco), 10% fetal bovine serum (FBS, Corning, NY, USA) to maintain tissue viability and prevent contamination [43]. Excess connective tissues were removed, and the eyeballs were disinfected using a 1:2 (v/v) solution of povidone-iodine and phosphate buffered saline (PBS, Gibco, Waltham, MA, USA) for 5 minutes. Following disinfection, the eyes were rinsed with sterile PBS.

A 30 mL of GV-expressing cell mixture at different concentrations (2 × 10 ^7^ and 1 × 10 ^7^ cells/mL) was injected into the subretinal space. The subretinal injection was performed following a published protocol [44]. Briefly, the eyeball was secured in a customized holder under a dissecting microscope. The superior rectus muscle was excised using sterile scissors, and a scleral tunnel was created 3.5 mm posterior to the limbus using a 26-gage sterile disposable needle. A contact lens coated with Gonak gel (Akorn Inc., Lake Forest, IL, USA) was placed on the cornea to stabilize the ocular surface and visualize the injection site. Using a 30-gauge Hamilton syringe loaded with the cell suspension, the needle was carefully introduced through the scleral tunnel and advanced under microscopic guidance until it approached the retinal space. The cell suspension was slowly injected with care to avoid disruption to the retinal tissue. After the injection, the syringe was gradually retracted to minimize the risk of tissue damage.

*Imaging Systems*: For in vitro and ex vivo imaging, a spectral domain optical coherence tomography (SD-OCT, TEL321, Thorlabs, Newton, NJ, USA) system modified with an ophthalmic lens (AC080-010-A) was used [45, 46]. SD-OCT is a reliable and non-invasive imaging technique that employs infrared light to detect backscattered photons by low-coherence interferometry, allowing high-resolution analysis of the anatomical structures of the eye.

The OCT system employed an illumination light source with a center wavelength of 1300 nm. The light beam was split into reference and sample arms. In the reference arm, reflected light was directed back into the light source fiber, with the intensity of the reference light adjusted using an iris. In the sample arm, the beam passed through a 2D galvanometer and was focused onto the sample using a scan lens (LSM03, Thorlabs) and ophthalmic lens. The light reflected from the sample was combined with the reference light in the interferometer to produce interference patterns, which were detected by a line scan camera operating at up to 146 kHz repetition rates. The system achieved lateral and axial resolution of 13.0 µm and 5.5 µm in air, respectively. For all OCT images, A-lines were acquired at 10 kHz. For in vitro studies, each B-scan consisted of 400 A-scans, with each A-scan sampled at 1024 pixels over an axial depth of 1 mm. For ex vivo studies, each B-scan consisted of 845 A-scans, with each A-scan sampled at 1024 pixels, corresponding to a field of view (FOV) of approximately 5.49 mm (lateral, X) by 2.96 mm (axial, Y). Each pixel corresponds to approximately 5.36 µm in the lateral direction and 3.50 µm in the axial direction.

*Data analysis*: The OCT data were acquired through ThorImageOCT software (Thorlabs), and images were processed with MATLAB (Mathworks Inc., Natick, MA, USA) and ImageJ (National Institutes of Health, Bethesda, Maryland, USA). The statistical analysis was completed with GraphPad Prism 10 (GraphPad Software Inc., San Diego, CA, USA).

## Electronic supplementary material

Below is the link to the electronic supplementary material.

Supplementary material 1

Supplementary material 2

Supplementary material 3

Supplementary material 4

## Data Availability

The data sets obtained and used in this study are available upon request submitted to the corresponding author (Lian@psu.edu).
