# Supplementary figures and images for: Gas vesicle-expressing human pluripotent stem cells enable multimodal ultrasound and optical coherence tomographic imaging
Source: BMC Biotechnol. 2026 Apr 30;26:78. doi: 10.1186/s12896-026-01161-x (PMC13274054; doi:10.1186/s12896-026-01161-x)

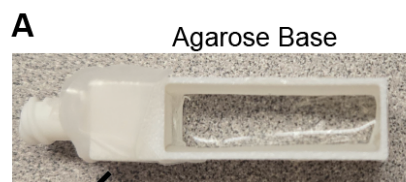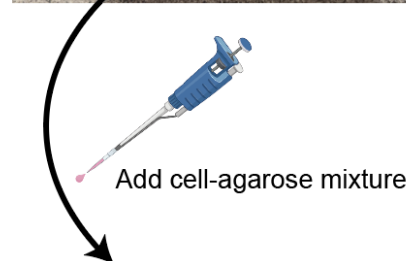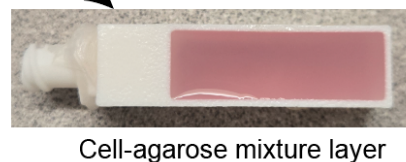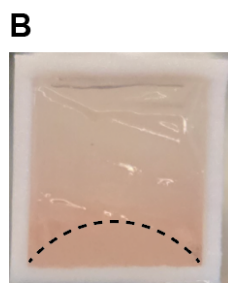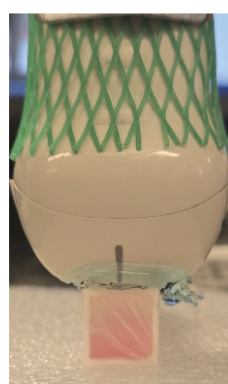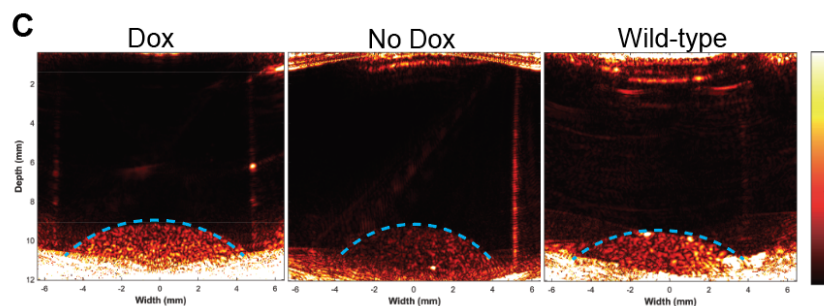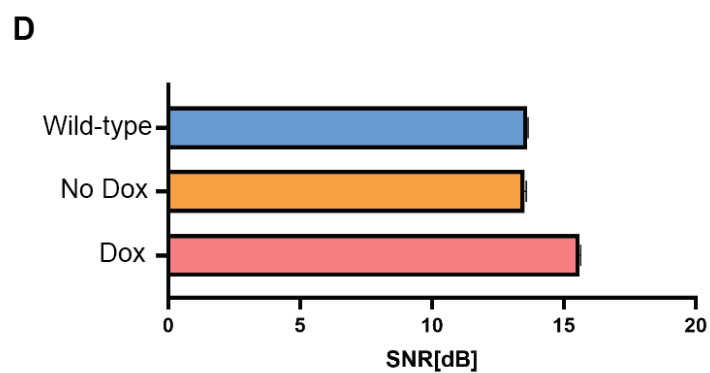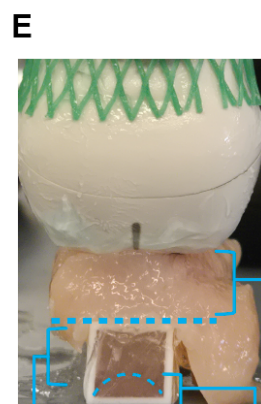

Chicken Breast  
Agarose Only  
Cell-Agarose Mixture

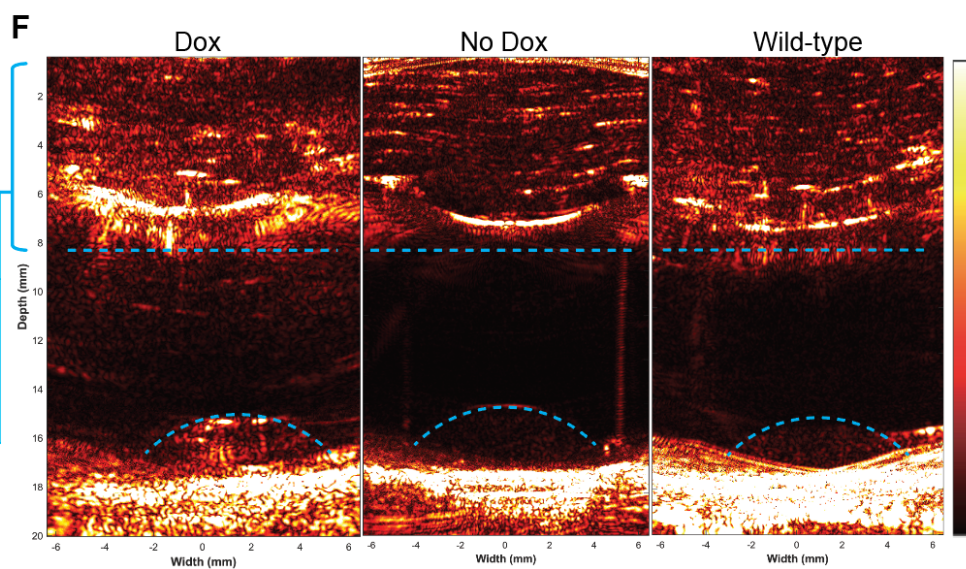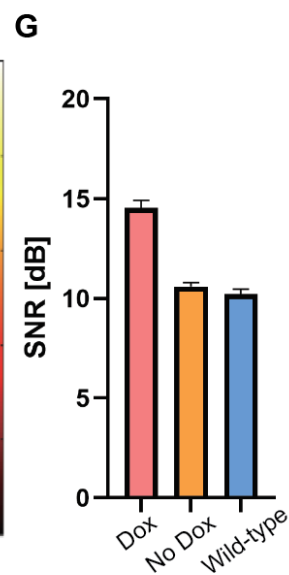

Supplement: Supplementary file 1 — Supplementary material 1 [file 12896_2026_1161_MOESM1_ESM.pdf]
