# Supplementary material for: Gas vesicle-expressing human pluripotent stem cells enable multimodal ultrasound and optical coherence tomographic imaging
Source: BMC Biotechnol. 2026 Apr 30;26:78. doi: 10.1186/s12896-026-01161-x (PMC13274054; doi:10.1186/s12896-026-01161-x)
Supplement: Supplementary file 2 — Supplementary material 2 [file 12896_2026_1161_MOESM2_ESM.docx]

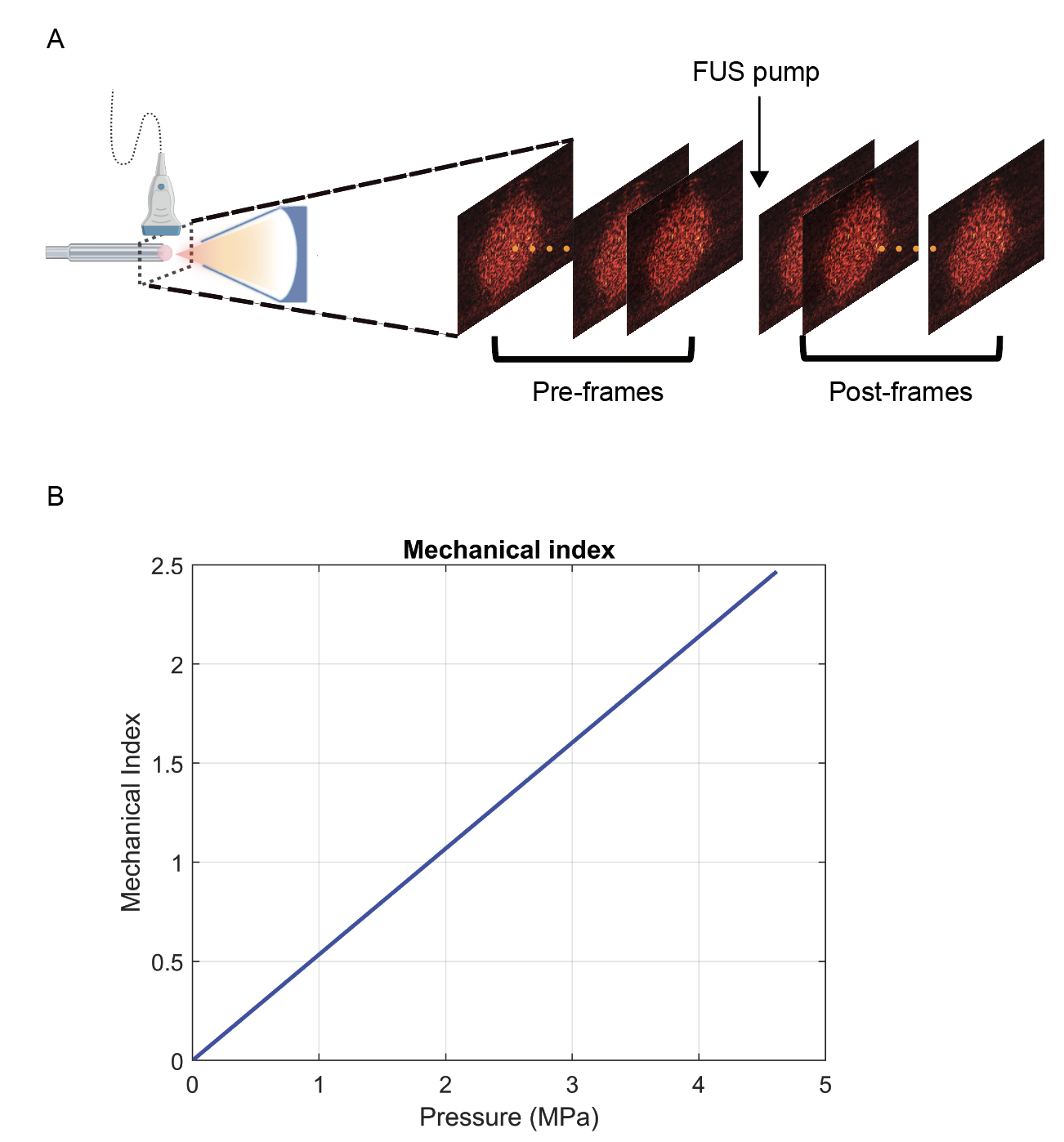


**Figure S1**. A) Schematic representation of the experimental protocol for frame acquisition before and after focused ultrasound (FUS) exposure. Agarose mixed cells were imaged using high-frame-rate ultrasound imaging before and after the application of a single FUS pulse. The FUS was delivered at the center of the imaging region. Pre-FUS frames were acquired immediately prior to FUS exposure, while post-FUS frames were acquired following the pulse. The highlighted regions represent frames used for pre-FUS and post-FUS analysis. B) Mechanical index (MI) as a function of peak negative pressure used during FUS stimulation. This linear relationship illustrates the range of mechanical indices corresponding to the pressures applied during the experiment (0–5 MPa. The MI was calculated using the standard formula where peak negative pressure in MPa, and f is the transmit center frequency in MHz (assumed to be 3.5 MHz in this study), $MI=P_{neg}/\sqrt{f}$*.*

**Supplementary video 1 to 4**. Representative OCT images of agarose phantoms with GV-expressing hPSCs. Video of B-scan images at concentrations of 5 x 10^6^ cells/mL (video 1), 1 x 10^6^ cells/mL (video 2), 5 x 10^5^ cells/mL (video 3), and 1 x 10^5^ cells/mL (video 4).
