# Supplementary material for: Gas vesicle-expressing human pluripotent stem cells enable multimodal ultrasound and optical coherence tomographic imaging
Source: BMC Biotechnol. 2026 Apr 30;26:78. doi: 10.1186/s12896-026-01161-x (PMC13274054; doi:10.1186/s12896-026-01161-x)
Supplement: Supplementary file 4 — Supplementary material 4 [file 12896_2026_1161_MOESM4_ESM.docx]

**Supplementary Figure S1**: *Ex vivo* deep-tissue ultrasound imaging of GV-expressing hPSCs. A) Schematic illustration of the step-by-step preparation of the bi-layered sample in a custom cuvette. A blank agarose layer is first cast and solidified, followed by the addition of a cell-agarose mixture to create a distinct two-layer structure. B) Cross-sectional photographs of the fully assembled cuvette sealed with an acoustically transparent membrane. The separated blank agarose and cell-agarose layers are clearly visible. C) *In vitro* ultrasound B-mode images of the cuvettes shown in (B). This baseline imaging demonstrates the distinct echogenic differences between the layers before tissue insertion. D) Quantitative analysis of the Signal to Noise Ratio (SNR) for the *in vitro* ultrasound images, comparing Dox, No Dox and Wild-type samples. E) *Ex vivo* experimental setup. The prepared bi-layered cuvette is embedded beneath a layer of fresh chicken breast tissue to simulate a deep tissue environment. F) Ex vivo ultrasound B-mode images acquired through the chicken breast tissue for Dox, No Dox, and Wild-type groups. G) Corresponding quantitative SNR analysis for the *ex vivo* ultrasound images.
